# External Surface Quality of the Graphite Crystallizer as a Factor Influencing the Temperature of the Continuous Casting Process of ETP Grade Copper

**DOI:** 10.3390/ma14216309

**Published:** 2021-10-22

**Authors:** Paweł Kwaśniewski, Paweł Strzępek, Grzegorz Kiesiewicz, Szymon Kordaszewski, Krystian Franczak, Michał Sadzikowski, Wojciech Ściężor, Anna Brudny, Joanna Kulasa, Barbara Juszczyk, Romuald Wycisk, Michał Śliwka

**Affiliations:** 1 Faculty of Non-Ferrous Metals, AGH University of Science and Technology, 30-059 Krakow, Poland; kwas@agh.edu.pl (P.K.); gk@agh.edu.pl (G.K.); szyk@agh.edu.pl (S.K.); kfranczak@agh.edu.pl (K.F.); msa@agh.edu.pl (M.S.); wsciezor@agh.edu.pl (W.Ś.); 2 The Department of Advanced Material Technologies, Łukasiewicz Research Network—Institute of Non-Ferrous Metals, 44-121 Gliwice, Poland; anna.brudny@imn.gliwice.pl (A.B.); joanna.kulasa@imn.gliwice.pl (J.K.); barbara.juszczyk@imn.gliwice.pl (B.J.); 3 Carbo-Graf Sp. z o.o., 47-400 Raciborz, Poland; wycisk@carbograf.pl (R.W.); michal.sliwka@carbograf.pl (M.Ś.)

**Keywords:** continuous casting, Cu-ETP, FEM analysis, casting parameters, process temperature, surface quality, crystallizer, graphite

## Abstract

Today’s world is a place where lack of electrical energy would be unimaginable for most of society. All the conductors in the world, both aluminum and copper, have their origin in various types of casting lines where the liquid metal after crystallization is being processed into the form of wires and microwires. However, the efficiency of the continuous casting processes of metals and the final quality of the manufactured product strictly depend on the design of the used crystallizers, the materials used during its production and its quality. Research conducted in this paper focuses on the latter, i.e., external surface quality of the graphite crystallizer at the place of contact with the primary cooling system. In order to quantify its influence on the continuous casting process numerical analyses using the finite element method has been conducted, which results have been further confirmed during empirical tests in laboratory conditions. It has been proven with all of the proposed methods that the temperature of the obtained cast rod is closely linked to the aforementioned surface quality, as when its roughness coefficient surpasses a certain value the temperature of the obtained product increases almost twofold from approx. 150–170 °C to 300–320 °C. These values might influence the quality and final properties of the cast rod, the susceptibility to wire drawing process and possible formation of wire drawing defects and therefore be of much importance to the casting and processing industry.

## 1. Introduction

As the world progresses and develops the demand for electrical energy increases in a drastic rate which makes it necessary to improve the processing of electrical conductive materials. Knowing that copper is second only to silver in terms of electrical conductivity makes it an essential material in electrical wiring and transportation. It has a significant role in alternative energy sources such as wind turbines or solar panels, but it is also a crucial element of the electric vehicles as it requires four times more copper than internal combustion engine cars [1,2]. Before copper may be processed into wires or microwires it has to undergo the process of casting, usually in one of the continuous systems, as it may be casted horizontally [3,4,5] or vertically [6,7,8] in both directions (up or down). Each of the processes has its advantages and disadvantages in terms of the properties of the final product; however, all of them are used all over the world to produce copper wire rods and cast rods [9]. Many aspects of the continuous casting processes these days are being simulated using finite element method. It of course does not reflect fully the process, however, it may help to understand previously unknown variables such as temperature field [10], solidification [11] and solidification in the vacuum [12,13], fluid dynamics [14] or electromagnetic field and electromagnetic stirring [15,16,17,18]. The latter has been also extensively tested in the laboratory conditions and further on the industrial scale as electromagnetic stirring as it has been proven that it enables to improve the final product quality and the productivity of the process [19,20] and, what is more the as the authors in [21] claim the formerly inhomogeneous columnar grain structure turns into homogeneous equiaxed grain structure with the application of alternating magnetic field.

The essence of the continuous casting process lies in the continuous supply of liquid metal to the primary cooling system where the liquid metal crystallizes into a cast rod or strand. The crystallization system, which is most often made of a graphite insert, the so-called crystallizer, and a cooling system perform the essential function in this process as it is responsible for the heat transfer during the process [22,23]. The effectiveness of such system essentially depends on the characteristics of the materials used for the crystallizer and the cooling system, their geometry and the quality of the combination of these two structures. Crystallizers in the continuous casting process, especially copper and its alloys, and at much lower rate precious metals and their alloys, are usually made of graphite-based materials. One of their most important features is the fact that these graphite materials are hardly wettable by copper and, moreover, have high thermal conductivity and self-lubrication properties [24,25,26,27,28]. A different group of crystallizers are solutions used in continuous casting systems appropriated for steel. These are metal crystallizers made of alloy copper sleeves, most often with a chromium platter with a specially developed internal surface [29]. A characteristic feature of metal crystallizers is the use of an additional medium that separates the cast metal from the crystallizer, which may be loose solids, liquid or gas. However, usually various greases are used as when subjected to high temperature during the process the grease evaporates leaving at the surface of the crystallizer only carbon black, which provides the same properties as self-lubricating graphite crystallizer. It has been calculated and proven that the power received from the solidifying material by the crystallizer or mould is being determined by the chemical composition of the material and therefore its physical properties [30,31], temperature of the liquid metal and final ingot temperature and size [32], the feed of the process [33,34] and parameters, geometry and temperature of the crystallizer during the process [35,36,37]. However, the influence of the external surface quality of the crystallizer on the temperature of the crystallizer, and therefore on the final product, has not yet been determined, even though, the contact quality of the crystallizer with the cooling system may have a significant impact on the temperature of the cast rod or strand. The continuous casting processes of metals develop every day and even though, old challenges remain and new ones appear, every method of minimizing casting defects, increasing the quality of the product and making it possible to cast alloys that could originally be casted only via other means makes an important contribution to the industrial production [38]. Research proposed in this paper is crucial for the continuous casting lines in which the final product is a cast rod (unlike wire rod like in continuous casting and rolling lines) and the crystallization system uses graphite crystallizers like for instance Rautomead or Upcast. The research regarding the external surface of the graphite crystallizer and its influence on the temperature distribution during the process of continuous casting of electrolytic tough pitch (ETP) grade copper has been divided into two main parts, i.e., finite element method (FEM) simulations and empirical research in laboratory conditions.

## 2. Materials and Methods

**Figure 1:**
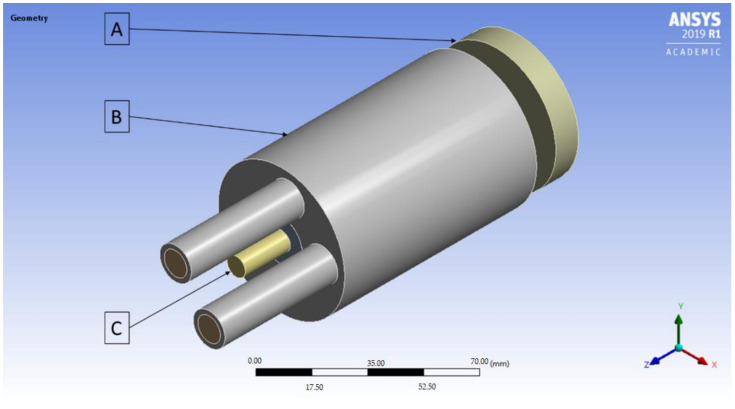
A general view of the implemented during the FEM simulations crystallization system; (**A**)–a graphite crystallizer, (**B**)–a copper cooling system, (**C**)–a copper cast rod.

**Figure 2:**
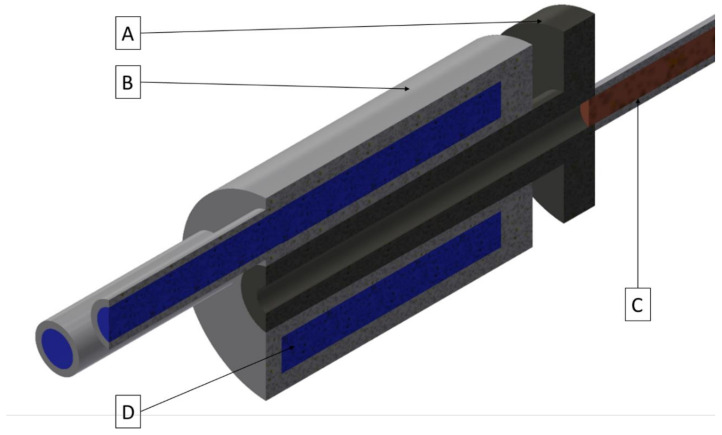
A cross-section of the crystallization system; (**A**)–a graphite crystallizer, (**B**)–a copper cooling system, (**C**)–a copper cast rod, (**D**)–cooling medium.

The conducted research began with the FEM simulations of the continuous casting process using the ANSYS Workbench software with FLUENT Fluid Flow module (2019 R1 version 19.3.0, Ansys, Canonsburg, PA, USA). The temperature distribution throughout the process with the distinction of the temperature of the cooling medium after exiting the cooling system, temperature of the copper cooling system, temperature of the graphite crystallizer and temperature of the cast rod have been determined. The applied variable was the external surface quality of the graphite crystallizer at the place of contact with the cooling system ranging from Ra 0.08 μm to Ra 5 μm (to be precise 0.08, 0.16, 0.32, 0.64, 1.25, 2.5 and 5) and additional diversification of the crystallizer–cooling system fitting for Ra 1.25 μm variant (loose fit, medium fit and tight fit). The temperature of the liquid metal during the process has been set to 1250 °C with medium velocity 0.5 L/min, casting feed of 1 mm/s and the diameter of the cast rod of 8 mm. The process was simulated in 1000 steps every 0.5 s each. A general view of the implemented crystallization system during the FEM simulations have been presented in Figure 1 and the cross-section has been presented in Figure 2.

**Figure 3:**
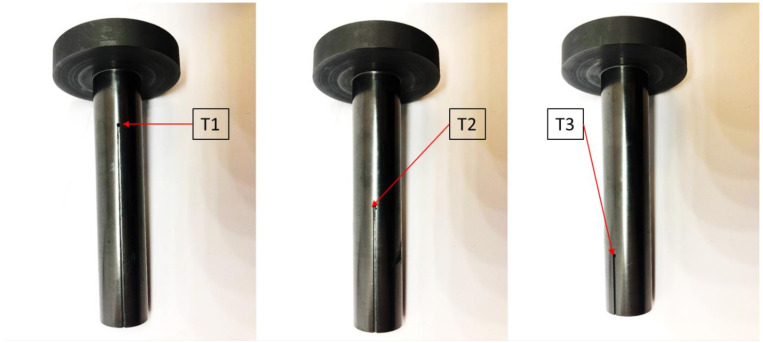
Graphite crystallizer with 3 points of measurement (T1)–at the beginning of the crystallizer, (T2)–in the middle of the crystallizer, (T3)–at the end of the crystallizer.

An empirical part of the research was conducted in laboratory conditions using graphite crystallizers (Carbo-graf sp. z o.o., Racibórz, Poland) during actual vertical continuous casting process. The process was conducted using an especially prepared prior casting graphite crystallizer with 3 measuring points every 120° in which thermocouples (Czaki Thermo-Product, Rybie, Poland) were inserted in order to constantly measure the temperature of the crystallizer as presented at Figure 3.

**Figure 4:**
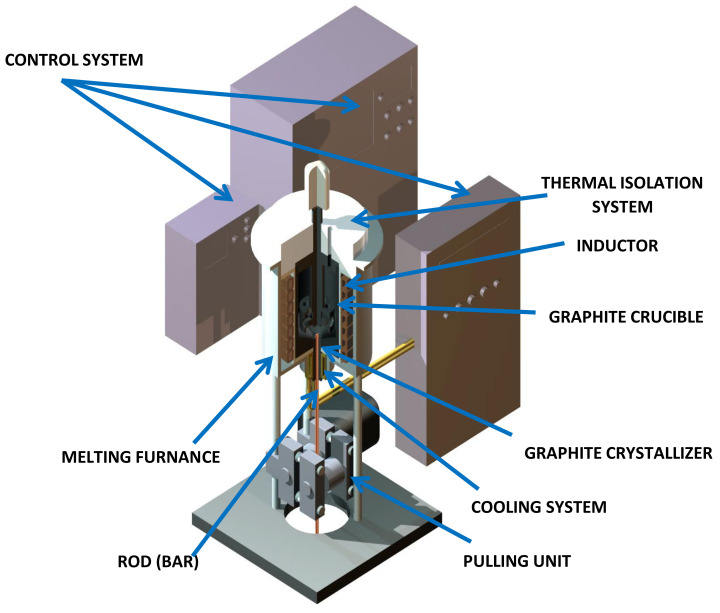
Schematics of the melting and casting furnace.

The actual continuous casting process in laboratory conditions was conducted using the melting and casting furnace (Termetal, Piekary Śląskie, Poland) presented schematically at Figure 4. The nominal power of the furnace is 20 kW and the frequency of the induction coil is 3 kHz. Before the conducted processes 3 types of graphite crystallizers were prepared, with the surface roughness of Ra 0.2 μm, Ra 2.3 μm and Ra 5 μm. Besides the temperatures of the crystallizers, the temperature of the cast rod and the cooling medium before and after exiting the crystallization system was recorded. The tests were conducted with various parameters ranging in terms of both the casting feed (between 1 mm/s and 50 mm/s) and velocity of the cooling medium (between 0.5 L/min and 2.2 L/min). The wide spectrum of the parameters made it possible to conduct a thorough analysis of the existing problem in terms of the contact quality between crystallizer and cooling system.

**Figure 5:**
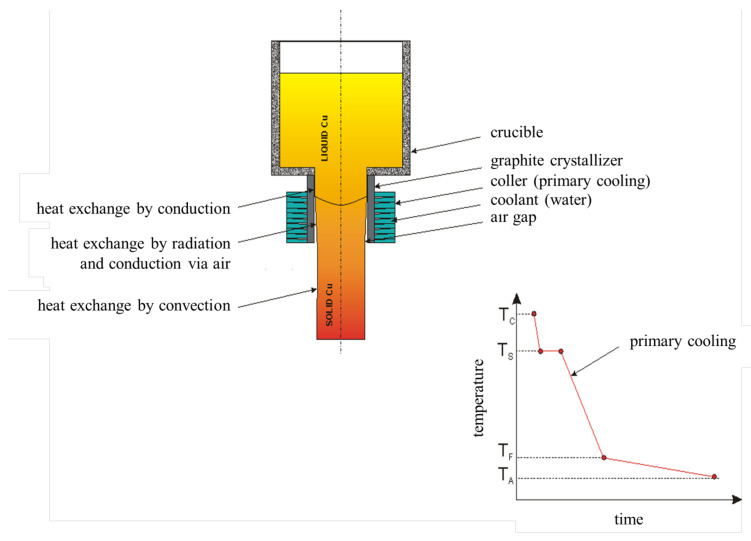
Schematics of the heat exchange during the continuous casting process; T_C_–casting temperature, T_S_–solidification temperature; T_F_–final cast rod temperature (after exiting the crystallizer); T_A_–ambient temperature.

Schematics of the heat exchange during the continuous casting process and the cooling line throughout the process are presented in Figure 5. The pure metal solidifies in the constant temperature so there is an observable stop on the cooling line and good contact between metal and crystallizer provides cooling via conduction. After solidification, a continuous decrease of cast temperature is observable down to the ambient temperature. Firstly, in the crystallizer by conduction where, due to the contact between cast and crystallizer, the heat exchange takes place and secondly, by radiation and air conduction and thirdly after exiting the crystallizer by convection. In many practical continuous casting processes there is an opinion that radiation controls heat flow inside crystallizer. It is true in the case of big diameter billets. In the studied case where the diameter is 8 mm, the air gap is very small, and conduction via air may be significant even despite a small thermal conductivity of air. There is no secondary cooling system installed, so the casting feed is limited; however, there is no direct contact between hot cat rod and cold cooling medium and therefore there are no high thermal stresses making it a good way of simulating the processes like Upcast of Rautomead.

## 3. Results and Discussion

Research works conducted in order to determine whether the external surface roughness of the crystallizer at the place of contact with the cooling system throughout the continuous casting process of ETP grade copper was divided into two parts. Firstly, using FEM simulations with various crystallizer qualities and steady process parameters, and secondly, after the range values were determined, three types of crystallizer qualities were selected for the actual continuous casting process in various conditions. The proposed simulations and experimental procedures may help to answer the question regarding the impact of the quality of contact between a crystallizer and a cooling system throughout the continuous casting processes. Even though the process was conducted on a laboratory scale, the actual industrial conditions may show similar dependencies, even though the casting feed is much greater; however, so is the velocity of the cooling medium.

### 3.1. Finite Element Method Simulations of Continuous Casting Process

**Figure 6:**
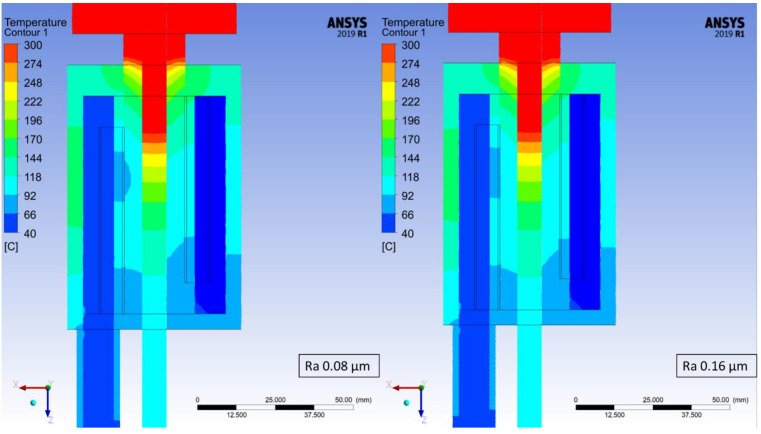
Continuous casting process simulation for the crystallizer with Ra 0.08 μm (**left**) and Ra 0.16 μm (**right**); step 900 of the simulations (450 s).

**Figure 7:**
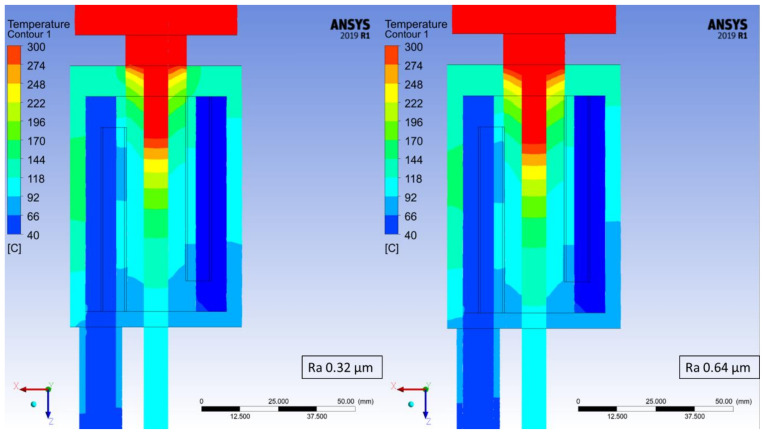
Continuous casting process simulation for the crystallizer with Ra 0.32 μm (**left**) and Ra 0.64 μm (**right**); step 900 of the simulations (450 s).

**Figure 8:**
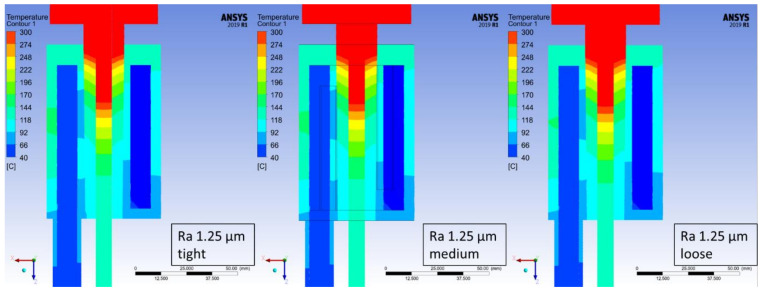
Continuous casting process simulation for the crystallizer with Ra 1.25 μm and tight fit with the cooling system (**left**), medium fit with the cooling system (**middle**) and loose fit with the cooling system (**right**); step 900 of the simulations (450 s).

**Figure 9:**
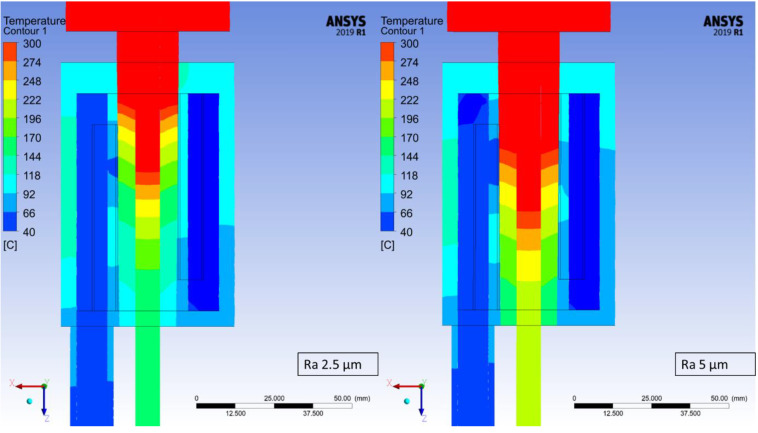
Continuous casting process simulation for the crystallizer with Ra 2.5 μm (**left**) and Ra 5 μm (**right**); step 900 of the simulations (450 s).

The temperature distribution has been determined for each of the studied cases based on the maximum temperature at every measuring point. At the worst studied case (crystallizer with the highest roughness coefficient) it took 870 steps for the process to stabilize in terms of the recorded temperatures and therefore, step number 900 was chosen from each of the simulations in order to depict the changes in recorded temperatures, which are presented in Figure 6, Figure 7, Figure 8 and Figure 9. Each Figure represents the process with the same parameters in terms of casting feed, temperature of the liquid metal, velocity and temperature of the cooling medium with only one variable, the external surface quality of the crystallizer.

The maximum values at the selected measuring points of the above Figure 6, Figure 7, Figure 8 and Figure 9 were collectively presented in Table 1. Firstly, in order to determine the prospective influence of the fitting between the graphite crystallizer and the cooling system, 3 various cases were simulated with the Ra 1.25 μm (tight, medium and loose), whereas for all the other cases, the fitting was medium. There has been little to no effect recorded in terms of the temperature distribution for various fitting types. The temperature of the cast rod dropped by approximately 3 °C when the fitting was tight and rose by approximately 6 °C when the fitting was loose, both in comparison to the medium fitting. The temperature of the cooling medium and the cooling system remains at a steady level and the temperature of the crystallizer changes similarly to the cast rod temperature. The situation is drastically different when the changes in surface quality are considered. The cooling medium and the cooling system temperature drop is quasi-linear, meaning that as the contact quality between the crystallizer and the cooling system deteriorates, the heat exchange worsens as well. It is clearly visible when the temperature values of the crystallizer and the cast rod increase significantly, even by 100% when comparing the best (crystallizer 97.57 °C and cast rod 167.71 °C) and the worst (crystallizer 228.08 °C and cast rod 317.55 °C) surface qualities tested. The changes are also clearly visible in Figure 6, Figure 7, Figure 8 and Figure 9, where the process is always at the same stage after the temperatures have stabilized, the parameters are exactly the same, and the colour of the cast rod (and by contact also the colour of the crystallizer) undoubtedly changes from blue, to greenish, green, and finally yellow, suggesting that the temperature rise is unquestionable. Data as a whole suggest that the heat exchange from the cast rod, through the crystallizer to the cooling system throughout the process, is being compromised by the worsening contact quality to the point that there is a visible, significant difference in temperature distribution. It is worth mentioning that the recorded increase up to Ra 0.63 μm is not that meaningful, as it only rises by less than 20 °C (cast rod) and 12 °C (crystallizer).

Data obtained using finite element method simulations were the starting point for the actual continuous casting process conducted in laboratory conditions. As it turns out there is no significant change in temperature values for the better surface qualities of the crystallizers, and therefore, the process was conducted using only the 3 graphite crystallizers i.e., with the best possible to obtain surface quality of Ra 0.2 μm (representing extremely good surface quality), medium surface quality of Ra 2.3 μm (representing the commercial quality obtained with turning lathe) and the worst surface quality of Ra 5 μm (representing extremely bad surface quality).

### 3.2. Continuous Casting Process in Laboratory Conditions

There are many variables during the continuous casting process of metals, and taking even one out of the equation is a huge step forward towards the failure-free technology. Usually there is no attention given to the external surface quality of the graphite crystallizer, as there were no data proving that it has a significant value. That is why the simulation is not enough and extensive experimental verification of the obtained results have been conducted with the use of the exact same continuous casting system as it was implemented in the FEM simulation software.

The obtained results are the mean values of the measurements recorded with the use of precisely placed thermocouples, which made it possible to provide a vast amount of information regarding temperature distribution. In Table 2 the data are presented in groups marked with 5 different colours; each represents a different set of process parameters (feed and standstill). In each of the studied cases, as the roughness coefficient rises, the rise in the temperature of the cast rod and each of the 3 measuring points of the crystallizer occurs regardless of the parameters of the process. Of course, as the feed increased or the standstill decreased, the velocity of the cooling medium had to be modified appropriately and therefore giving quite similar conditions each time. The measured temperature of the cast rod for extremely good quality of the crystallizer surface (Ra 0.2 μm) is always around 160–190 °C; when considering the medium surface quality (Ra 2.3 μm) the range is between 220 °C and 250 °C, and finally when taking into account the extremely bad surface quality (Ra 5 μm), the range is the broadest as the measured temperatures between 275 °C and 415 °C. Even though it may seem that the range of the recorded values is enormous, especially in the case of the last discussed type of crystallizers, it undoubtedly shows a growing tendency as the surface quality impairs. Temperature of the crystallizer also deserves further analysis as it clearly shows, that when considering the 3 measuring points, the significant difference is visible between T1 and T2 (the beginning of the crystallizer and the middle of the crystallizer) whereas there is almost no difference between points T2 and T3 (end of the crystallizer), even though the measuring points were placed at the exact same distance from each other. However, again the measured crystallizer temperatures increase drastically as its surface quality worsens. On the other hand when considering the water temperature the important part is to remember that the better the heat exchange during the process the higher will be the temperature of the water when it will exit the cooling system. Therefore, when the surface quality of the crystallizer is the best the water temperature is the highest as the cooling process is being carried out in the best conditions.

In Table 3 the comparison of the obtained results of the finite element method simulation with the actual continuous casting process in laboratory conditions with corresponding parameters was presented. The experimental procedure had a purpose of verifying the prior obtained data. When put together it is obvious that the differences are minimal (less than a few %) in terms of the temperature of the cast rod, and the temperature of the crystallizer from the simulation is similar to the value measured at the middle of the crystallizer during the experimental part.

## 4. Conclusions

Taking into consideration the obtained FEM simulations, empirical research results and conducted calculations, the following may be stated:

1. The external surface quality of the graphite crystallizer at the place of contact with cooling system above a specific roughness coefficient value is of much importance in terms of temperature distribution during the continuous casting process of copper and possible other metals. The obtained finite element method simulations results show that there are little to none differences up to Ra 0.63 μm and a small difference at 1.25 μm in comparison to Ra 0.08 μm, with a significant influence of the surface quality when it is equal or worse than Ra 2.5 μm.
2. The conducted FEM simulations reflected the experimental research carried out later on. When put together, the differences are minimal (less than a few %) considering the temperature of the cast rod, whereas the temperature of the crystallizer from the simulation is similar to the value measured at the middle of the crystallizer during the experimental part in corresponding conditions.
3. The continuous casting process conducted in laboratory conditions proved that the measured temperature of the cast rod for extremely good quality of the crystallizer surface (Ra 0.2 μm) is always around 160–190 °C and 170.6 °C for the FEM simulation, when considering the medium surface quality (Ra 2.3 μm) the range is between 220 °C and 250 °C and 244.69 °C for the FEM simulation, and finally when taking into account the extremely bad surface quality (Ra 5 μm) the measured range is between 275 °C and 415 °C and 317.55 °C for the FEM simulation.
4. The better the external surface quality of the crystallizer, the better the heat exchange from the liquid metal and the possibility of obtaining a higher casting efficiency, which is the essence of this research work. Most often, the desired efficiency of the cooling process is as high as possible, which allows for quick crystallization and cooling of the cast rod or strand and that requires high efficiency of heat flow, which may be achieved by using crystallizers with high surface quality (low roughness) and by the correct fit of the crystallization system.

## Figures and Tables

**Table 1:** Collective measurements data recorded during the finite element method simulations of the continuous casting process.

Crystallizer Variant	Ra 0.08 Medium Fit	Ra 0.16 Medium Fit	Ra 0.32 Medium Fit	Ra 0.63 Medium Fit	Ra 1.25 Tight Fit	Ra 1.25 Medium Fit	Ra 1.25 Loose Fit	Ra 2.5 Medium Fit	Ra 5 Medium Fit
Cooling medium (°C)	48.84	48.63	48.3	47.35	47.13	46.98	46.69	45.05	42.20
Cooling system (°C)	93.83	94.15	94.08	92.79	89.64	89.52	89.31	87.56	82.68
Crystallizer (°C)	97.57	99.32	103.03	109.33	120.31	122.45	127.34	157.47	228.08
Cast rod (°C)	167.71	170.60	176.30	186.14	201.76	204.44	210.40	244.69	317.55

**Table 2:** Collective data measured throughout the laboratory continuous casting process.

Crystallizer Variant		Casting Parameters		Cooling Medium			Crystallizer Temperature			Metal Temperature	
Cast Number	Ra	Feed	Standstill	Velocity	Temperature (Initial)	Temperature (Final)	T1	T2	T3	Liquid	Cast Rod
	(μm)	(mm)	(s)	(L/min)	(°C)						
1_1	0.2	5	0.3	0.9	19.6	55	234	93	81	1364	190
2_1	2.3	5	0.3	1.1	21.6	45.5	275	171	164	1350	220
3_1	5	5	0.3	0.9	18.9	43.6	464	262	265	1350	322
3_8	5	5	0.3	0.7–0.8	19.4	46.7	472	267	271	1253	385
1_2	0.2	5	0.2	1.7	19.2	52	195.3	68.3	64.7	1360	180
2_2	2.3	5	0.2	0.9	21.5	47.5	279	176	170	1360	238
3_2	5	5	0.2	0.9	18.9	45.5	475	267	275	1350	362
3_9	5	5	0.2	0.8	19.3	48	477	273	284	1250	390
1_3	0.2	5	0.1	1.6	19.2	54	220	81	68	1367	195
1_6	0.2	5	0.1	1.1	19.1	58	250	95	80	1350	203
2_3	2.3	5	0.1	1.3–1.4	20.1	45.7	281	175	172	1350	249
2_5	2.3	5	0.1	2–2.2	20.8	36.5	262	166	162	1350	216
2_11	2.3	5	0.1	1.2	21.6	46	278	148	177	1300	225
2_12	2.3	5	0.1	1.1	21.4	45.3	271	143	173	1250	219
3_3	5	5	0.1	0.9	18.9	47	480	274	283	1350	401
3_5	5	5	0.1	2.2	18.5	30.3	433	252	246	1350	370
3_10	5	5	0.1	0.8	19.5	49.8	483	179.5	296	1250	415
1_4	0.2	3	0.3	1.3	18.8	49–50	222.7	78.6	72	1340	163
2_4	2.3	3	0.3	0.9	21.1	45.7	282	172	167	1350	223
3_4	5	3	0.3	0.9	18.9	41	451	262	261.2	1350	319
3_11	5	3	0.3	0.8	19.5	44.5	453	264	276	1250	356
1_5	0.2	1	1	0.5	19	50	193	70.6	67	1370	190–160
2_6	2.3	1	1	0.7–0.8	21.7	44.3	242	157	152	1350	248
3_6	5	1	1	0.7	19.3	39	412	238	232	1350	275
3_12	5	1	1	0.5	19.6	45.5	391	245	245	1250	307

**Table 3:** Comparison of the obtained data with the finite element method simulation results of the representative cases.

Crystallizer Variant		Casting Parameters		Cooling Medium			Crystallizer Temperature			Metal Temperature		Calculated Percent Error
Cast Number	Ra	Feed	Standstill	Velocity	Temperature (Initial)	Temperature (Final)	T1	T2	T3	Liquid	Cast Rod	
	(μm)	(mm)	(s)	(L/min)	(°C)							%
Continuous casting-Finite element method simulations												
Ra_0.16	0.16	1	1	0.5	15	53.6	99.32			1250	170.6	-
Ra_2.5	2.5	1	1	0.5	15	50.3	157.47			1250	244.69	-
Ra_5	5	1	1	0.5	15	47.5	228.08			1250	317.55	-
Continuous casting-Experimental data												
1_5	0.2	1	1	0.5	19	50	193	70.6	67	1370	190–160	6.21–11.37
2_6	2.3	1	1	0.7–0.8	21.7	44.3	242	157	152	1350	248	1.35
3_6	5	1	1	0.7	19.3	39	412	238	232	1350	275	13.4
3_12	5	1	1	0.5	19.6	45.5	391	245	245	1250	307	3.32

## Data Availability

Data available upon request.
